# Chronic Kidney Disease in Boys with Posterior Urethral Valves–Pathogenesis, Prognosis and Management

**DOI:** 10.3390/biomedicines10081894

**Published:** 2022-08-05

**Authors:** Richard Klaus, Bärbel Lange-Sperandio

**Affiliations:** Division of Pediatric Nephrology, Department of Pediatrics, Dr. v. Hauner Children’s Hospital, Ludwig-Maximilians University, Lindwurmstrasse 4, 80337 Munich, Germany; richard.klaus@med.uni-muenchen.de

**Keywords:** posterior urethral valves, lower urinary tract obstruction, chronic kidney disease, end-stage renal disease, bladder dysfunction, vesicoamniotic shunting

## Abstract

Posterior urethral valves (PUV) are the most common form of lower urinary tract obstructions (LUTO). The valves can be surgically corrected postnatally; however, the impairment of kidney and bladder development is irreversible and has lifelong implications. Chronic kidney disease (CKD) and bladder dysfunction are frequent problems. Approximately 20% of PUV patients will reach end-stage kidney disease (ESKD). The subvesical obstruction in PUV leads to muscular hypertrophy and fibrotic remodelling in the bladder, which both impair its function. Kidney development is disturbed and results in dysplasia, hypoplasia, inflammation and renal fibrosis, which are hallmarks of CKD. The prognoses of PUV patients are based on prenatal and postnatal parameters. Prenatal parameters include signs of renal hypodysplasia in the analysis of fetal urine. Postnatally, the most robust predictor of PUV is the nadir serum creatinine after valve ablation. A value that is below 0.4 mg/dL implies a very low risk for ESKD, whereas a value above 0.85 mg/dL indicates a high risk for ESKD. In addition, bladder dysfunction and renal dysplasia point towards an unbeneficial kidney outcome. Experimental urinary markers such as MCP-1 and TGF-β, as well as microalbuminuria, indicate progression to CKD. Until now, prenatal intervention may improve survival but yields no renal benefit. The management of PUV patients includes control of bladder dysfunction and CKD treatment to slow down progression by controlling hypertension, proteinuria and infections. In kidney transplantation, aggressive bladder management is essential to ensure optimal graft survival.

## 1. Introduction

**Figure 1:**
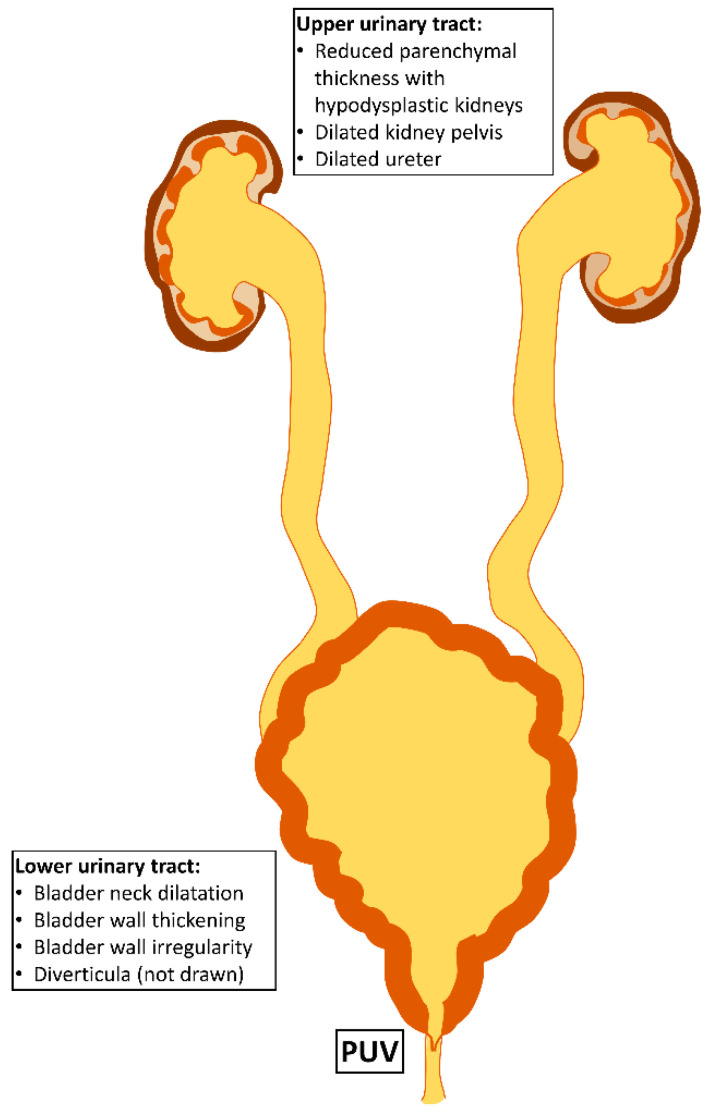
**Alterations in the urinary tract due to posterior urethral valves.** The subvesical obstruction in the prostatic urethra by posterior urethral valves (PUV) leads to a bladder neck dilation and increased urethral resistance, with the need for increased voiding pressure. As a consequence, bladder wall hypertrophy develops with the morphologic hallmarks of wall thickening and wall irregularity, possibly with diverticula. The obstruction can reach the upper urinary tract and dilate the ureter and the kidney pelvis. The functional tissue of the kidney, the parenchyma, can be reduced in thickness.

Posterior urethral valves (PUV) belong to the group of congenital anomalies of the kidney and urinary tract (CAKUT). CAKUT represent the major cause for end-stage kidney disease (ESKD) in childhood [1,2,3]. More specifically, PUV belong to a group of lower urinary tract obstructions (LUTO). These so-called valves, which are membranous sail-shaped structures in the lumen of the prostatic urethra, only occur in boys; they make up approximately 60% of all LUTO cases [4]. Subvesical obstruction leads to increased urethral resistance, which requires higher voiding pressures, causing bladder wall hypertrophy and deformation (Figure 1). If an obstruction reaches the upper urinary tract, it may impose pressure on the kidney pelvis with a reduction in parenchymal thickness. Kidney development is impaired in PUV patients, and may result in hypoplastic and dysplastic kidneys.

The total incidence of LUTO is about 3.3 per 10,000 pregnancies, and 2.3 per 10,000 live births [4]. This discrepancy emphasizes that LUTO is a potentially life-threatening disease for the fetus. In severe cases, oligohydramnion or anhydramnion impairs pulmonary development and leads to pulmonary hypoplasia, which is the major cause of mortality in boys with PUV, especially if it is present before the 20th week of gestation [5]. The incidence of PUV is about 1:3800 in live-born boys [6]. Historically, one third of the patients were diagnosed in the first month of life, another third in the first year of life and the last third thereafter [7]. The wide availability of prenatal ultrasound has made antenatal diagnosis possible and frequent. Approximately 35% of cases are diagnosed prenatally through typical findings with bilateral hydronephrosis and megacystis, key-whole sign and oligohydramnion or anhydramnion. Approximately 42% of cases are diagnosed in infancy, with weak urine flow and urinary tract infection being the leading symptoms. Twenty-three percent of the cases with rather mild phenotypes are detected in childhood through recurrent urinary tract infections (UTI), enuresis or bladder dysfunction [6]. Diagnosis after childhood is rare, and only reported in single case reports [8]. Postnatal management aims to ensure drainage, which can be achieved with a transurethral or suprapubic catheter. In severe cases, PUV patients develop polyuria and hyponatremia soon after catheter insertion. Therefore, it is necessary to monitor fluid status and electrolytes, in order to properly supplement sodium chloride if necessary and to assess kidney function and treat possible urinary tract infections (UTI). Primary valve ablation in the first 4 to 16 weeks of life through transurethral incision (TUI) is the preferred mode of management, along with administering urinary catheter drainage for at least the first 72 h [9]. The exact time point of TUI is also dependent on the size of the available resectoscope devices. In clinically unstable patients, e.g., with urosepsis, severe obstruction or in very low-weight preterm babies where primary valve ablation is technically difficult, drainage can be ensured by vesicostomy, ureterocutaneostomy or nephrostomy, and TUI can be delayed. Complications of TUI are rare, and unless urethral strictures occur the LUTO is usually removed completely and permanently. However, the impaired development of the kidneys and bladder is irreversible, and may result in lifelong complications such as bladder dysfunction and chronic kidney disease (CKD).

## 2. Pathophysiology of Chronic Kidney Disease in Posterior Urethral Valves

**Figure 2:**
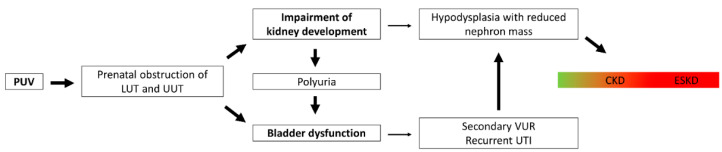
**Pathophysiology of chronic kidney disease in patients with posterior urethral valves.** Posterior urethral valves (PUV) lead to obstructions in the lower urinary tract (LUT) and the upper urinary tract (UUT), with consequent impairment of kidney development as well as bladder dysfunction. Impairment in kidney development may result in hypodysplasia with a reduced nephron mass, and can therefore cause chronic kidney disease (CKD). CKD can progress to end-stage kidney disease (ESKD). Bladder dysfunction can be aggravated by polyuria of the impaired kidneys, and cause secondary vesicoureteral reflux (VUR) and recurrent urinary tract infections (UTI). The latter may cause parenchymal scars in the hypodysplastic kidneys and accelerate CKD progression.

In children with PUV, two major factors contribute to the progression to CKD: impairment of nephrogenesis, and bladder dysfunction. These may persist even after transurethral valve incision. The main pathways from PUV to CKD are shown in Figure 2.

### 2.1. Intrauterine Obstruction Impairs Kidney Development

Human nephrogenesis is an intrauterine process that begins in the 5th week of gestation and is completed by the 34th–36th week of gestation [10]. It results in a nephron mass of approximately one million nephrons per kidney. In preterm newborns, nephrogenesis may continue postnatally to a maximum of three weeks, but results in structurally abnormal glomeruli [11,12,13]. This has two major implications: intrauterine damage to the developing kidneys in PUV patients is irreversible, and preterm babies have a lower nephron mass [14,15,16]. Over the course of life, even in healthy individuals, nephron mass and therefore kidney function is gradually lost; this loss may be accelerated by secondary factors such as acute kidney injury (AKI) of any cause (e.g., infection, dehydration, drugs or glomerulonephritis), or chronic processes such as hypertension or diabetes [17]. Once kidney function falls below certain thresholds, complications occur and need to be treated. Kidney replacement therapy (KRT) needs to be initiated in patients with ESRD. Therefore, a lower initial nephron mass in PUV patients is a major risk factor for the development of CKD, since those patients start this downward facing slope at a lower level [17].

PUV can manifest as early as the fifth week of gestation [10], and their appearance can result in subvesical obstruction, which causes developmental impairment in both the kidneys and the bladder (Figure 1). Older data from sheep as a PUV animal model suggest that mid-gestational subvesical obstruction induces fibrosis in the kidneys. The processes leading to this final pathway of fibrosis have been investigated in more detail in neonatal mice with unilateral ureteral obstruction (UUO). In mice, only 10% of glomeruli are formed at birth, with the remaining nephrons being generated postnatally. Based on the duration of nephrogenesis, the mouse at birth parallels the mid-trimester human fetus. Thus, UUO in the newborn mouse offers a suitable animal model to study obstructive uropathy. Like PUV, UUO leads to hydronephrosis, which imposes pressure on the renal parenchyma. This pressure induces various pathophysiological processes, such as activation of the renin angiotensin aldosterone system (RAAS), GFR loss and infiltration with inflammatory cells such as macrophages and T-cells [18,19,20]. Such cells secrete pro-inflammatory cytokines that lead to different modes of cell deaths, including apoptosis, necrosis and necroptosis [19]. Common final outcomes can be tubular atrophy, atubular glomeruli and interstitial fibrosis [18].

### 2.2. Bladder Dysfunction Results from Muscular Hypertrophy and Fibrotic Remodelling

Similarly with kidney development, bladder development is an intrauterine process that is impaired by LUTO. Normal bladder cycling develops in the third trimester, but is disturbed by the obstruction. Hence, postnatal valve ablation cannot ameliorate bladder dysfunction, which is very common in boys with PUV and occurs in 55% of cases [21,22]. There are few animal studies that investigated the pathophysiology of bladder dysfunctions. Most studies were performed in sheep using a mid-gestational gradual obstruction involving metal rings [23]. Bladder wall thickening with muscle hypertrophy and fibrosis were found in those animals. The degree of bladder fibrosis seems to be dependent on duration and severity of the obstruction [23]. Histologic samples to confirm those findings in patients are rare, but some data exist. In historic human samples from PUV bladders, muscular hypertrophy was dominant, with little or no fibrosis present [24,25]. However, in a very recent study with detrusor samples from children with PUV, increased expression of matrix metalloproteinase 7 and cyclin-D1 suggested that there was cellular remodelling with accumulation of extracellular matrix, including collagen and replacement of smooth muscle cells [26]. In addition to the fibrotic remodelling in the bladder, increased urethral resistance causes hypertrophy of the muscular bladder wall (Figure 1), which postnatally leads to a small capacity, hypercontractile bladder. All together, both hypertrophy and fibrotic remodelling contribute to postnatal contractile dysfunction in the PUV bladder. The polyuria caused by poor concentration ability of the dysplastic kidneys might contribute to worsening of bladder dysfunction and incontinence in the long run [27]. Bladder dysfunction also plays a role in CKD development, since it may lead to secondary reflux and recurrent UTIs. These are a potential threat for the remaining functional kidney tissue, since the inflammation can cause collateral damage with parenchymal scarring.

### 2.3. Genetic Causes of Posterior Urethral Valves

Even though there are relatively large genomic data sets, including more than 600 patients with LUTO, the only known monogenetic cause for anatomical LUTO are missense variants in basonuclin 2, a highly conserved zinc finger protein. The first missense mutation was identified in a family with a LUTO-phenotype across three generations, and consecutively three independent missense mutations were identified in other patients. Basonuclin 2 can be detected in lower urinary tract rudiments in human and mouse embryogenesis. It is expressed in the cytoplasm of urothelial cells, especially in the urogenital sinus, the precursor of the bladder and its outflow tract, but also in the urothelium of the male adult urethra. In functional studies, an experimental knockdown of basonuclin 2 in zebrafish caused pronephric-outlet obstruction and cloacal dilatation, phenocopying human congenital LUTO. In summary, those findings strongly implicate that basonuclin 2 missense variants are causative for LUTO phenotypes, even though the definitive function of basonuclin 2 is not fully understood [28].

## 3. Epidemiology of Chronic Kidney Disease in Posterior Urethral Valves

Recent reports from multicenter cohorts from the United States of 274 [29] and 685 [5] patients have shed new light on the epidemiology of CKD and ESKD in children with PUV. At the ages of 1, 5 and 15 years, 7%, 12% and 20% of children needed KRT, respectively [5,29]. In a smaller study with 35 infants, after 10 years 50% progressed to CKD stages 2–4, and 15% needed KRT [30]. Between 2002 and 2011, 11% of children with KRT in North America were as a result of PUV, which emphasizes the high socioeconomic burden of the disease [31]. Although the numbers are variable throughout the different reports, the portion of patients with CKD (up to 50%) and ESKD (up to 20%) are high enough to justify regular evaluation by a pediatric nephrologist for every boy with PUV.

## 4. Prognostic Factors for Chronic Kidney Disease Development

The possibility of stratifying the risk for CKD is highly relevant to guide parents and boys with PUV through the course of the disease, and to provide adequate medical and urological treatment, diet and frequency of follow-up visits. Renal prognoses can be estimated prenatally or postnatally. Therefore, the time point to estimate the renal prognosis should be discussed with the parents, taking into account their willingness to consider pregnancy termination. The important parameters used to estimate renal prognoses in the prenatal and postnatal periods are summarized in Table 1.

Even if parents do not want to have a prenatal estimate of the renal prognosis, severity of obstruction and especially the onset of anhydramnion should be monitored with prenatal ultrasound, since it has an important impact on pulmonary development and is essential for optimal preparation for postnatal respiratory care in the neonatal unit.

### 4.1. Prenatal Prognostic Factors

There have been debates over whether or not the time of diagnosis is an individual risk factor for CKD development. One argument is that an earlier manifestation will have a more severe phenotype; and on the other hand, an earlier diagnosis might prompt better treatment and hence improve renal outcomes. Sarhan et al. provided evidence for the latter through a large multicenter study, in which renal function was found to be better and upper urinary tract dilation milder in 315 boys with prenatal diagnoses [32]. However, two large recent studies that included 541 patients showed that there were no differences in renal outcomes between antenatal and postnatal diagnoses [33,34].

Prenatal ultrasound can be used to diagnose PUV and also to predict the severity of kidney involvement. In severe cases, signs of renal dysplasia can be seen prenatally and can predict a higher likelihood for CKD development. Those ultrasound findings include kidneys with small parenchymal area and parenchymal hyperechogenicity, cortical cysts and the absence of corticomedullary differentiation, all of which are associated with unfavourable renal outcomes. For the renal parenchymal area, a value below 8 cm^2^ in the third trimester was found to predict ESKD with a sensitivity of 71% and a specificity of 88% [35]; therefore, ultrasound has an even better predictive capacity than the so-called combined OCE, which includes oligohydramnion, cortical cysts and echogenic kidneys. Combined OCE predicts ESKD with a sensitivity of 47% and a specificity of 91% [36]. Another proposed ultrasound-based score used to predict impaired kidney function and perinatal mortality in fetuses with PUV stratifies the severity according to a combination of anhydramnion and bladder volume. Mild LUTO (no anhydramnion at the 26th week) had a risk of 11% for severely impaired kidney function (eGFR < 30 mL/min/1.73 m^2^), moderate LUTO (no anhydramnion before the 20th week of gestation, bladder volume > 5.4 cm^3^) a risk of 31%, and severe LUTO (anhydramnion before the 20th week of gestation, bladder volume > 5.4 cm^3^) had a risk of 44% for impaired kidney function [37].

The clinical application of fetal urine analysis is debatable as a result of the invasive nature of its collection and its lack of therapeutic consequences. However, many studies have analyzed fetal urine in order to predict CKD. Classical markers analyzed in fetal urine include electrolytes, osmolality and tubular proteins such as β-2-microglobulin. Increased sodium and chloride excretion, reduced concentration capacity with low osmolality and increased excretion of tubular proteins represent severe tubular damage as a result of the obstruction [38]. Based on these considerations, a combination of elevated β-2-microglobulin and elevated chloride in fetal urine was best in predicting CKD stages 4 and 5 in a follow-up time of 10–30 years, with an area under the curve of 0.89, a sensitivity of 93% and a specificity of 71% in 89 fetuses [39]. Proteomics has a major benefit in investigating proteins in biomaterials without defining specific targets prior to the analysis. Fetal urine accounts for the vast majority of peptides in the amniotic fluid, and therefore makes fetal urine an interesting biomaterial for assessing renal processes in the fetus [40]. The predictive capacity of fetal urine peptides is excellent and remains under active research. A panel of 12 peptides, called 12PUV, was defined and validated in order to prenatally predict renal failure in infancy. It consists of 12 peptide sequences that originate from different collagen-α-chains, and might therefore indicate the presence of fibrotic remodelling in the kidneys. In the recently published single-center validation study, early renal failure defined as eGFR < 60 mL/min/1.73 m^2^ in the first 6 months of life or perinatal death was predicted with an AUC of 0.89 and an accuracy of 84% [41,42]. These single-center findings are currently validated in the ANTENATAL trial, which aims to include more than 400 patients from > 30 European centers [43].

Another invasive prenatal method was described by Bajpai et al. Plasma renin activity in cord blood was higher in patients with early renal damage [44,45]. However, probably as a result of the invasive nature of the parameter, no data from other groups are available.

### 4.2. Postnatal Prognostic Factors

The predictive value of serum creatinine has been identified as a useful parameter in several studies in patients with PUV. Different time points from first month [46] of life, in the first year of life [47], days, weeks or years after ablation [29,48,49,50,51,52,53], have been investigated. Some studies also focused on the creatinine velocity [51,54], which assesses the change in creatinine over time. In conclusion, there is an abundance of data to support the hypothesis that nadir creatinine after valve ablation is the best independent predictor in infancy for the development of CKD in patients with PUV [29,48,49,50,51,52,53]. Although nadir creatinine was mostly used to stratify for only ESKD in older studies, recent studies with PUV patients have also proposed using cut-off values to predict different CKD stages. This has major clinical implications, since medical treatment usually begins at CKD stage 3, and therefore may change the daily lives of patients and families even though no KRT is required. These cut-offs values were deducted from 274 and 102 patients with PUV [29,48]. Both authors agree that a nadir creatinine >1.0 mg/dL is associated with a high risk of ESKD. In the cohort published by McLeod et al., all children that exceeded this threshold reached ESKD by the age of 8 years. Wu et al. reported the same threshold, with a specificity for ESKD over 90%. An older study proposed a more rigid cut-off at 0.85 mg/dL [50] to predict ESKD; however, because of a slight heterogeneity in endpoints, those values are not completely comparable. Historically, a cut-off of 0.8 mg/dL has been proposed by Warshaw et al. [55]. In conclusion, PUV infants with a nadir serum creatinine of 0.8 mg/dl are at very high risk, and should be monitored closely during childhood. On the other end of the spectrum, infants with a nadir creatinine below 0.4 mg/dL never reached ESKD until the age of 20 years [29]. This cut-off was also supported by Coleman et al. [50]. Data from Wu et al. point in the same direction, a 6-week nadir creatinine below 0.3 mg/dL was associated with a low risk of any CKD, and a value below 0.5 mg/dL was associated with a low risk for CKD stage 3 or more. Through serial measurements, Wu et al. added the information that nadir creatinine 6 weeks after valve ablation predicts renal function with similar accuracy as nadir creatinine after one year, and that both values are more accurate than serum creatinine at presentation. Therefore, the serum creatinine value 6 weeks after valve ablation achieves high accuracy and early prediction [48]. It is highly recommended to monitor the well-evaluated risk factor serum creatinine in all boys with PUV after valve ablation, in order to identify those at risk for severe CKD and ESKD. Even though nadir creatinine is an easily available parameter as well as the best independent prognostic marker, recent studies also propose scores that include multiple clinical parameters such as the presence of hypertension and proteinuria, in order to further improve stratification [56]. There is little data on serum markers other than for creatinine. Bajpai et al. described an increase in plasma renin activity as a potential marker for unfavourable renal outcomes in 58 patients; however, since 2013, no new data on this marker have been published [44].

The presence of bladder dysfunction is another important prognostic factor for ESKD in patients with PUV. Although three heterogeneously defined studies showed that bladder dysfunction is associated with ESKD, with relative risks between 1.15 and 8.9 [49,57,58]. Interestingly, the presence of bladder dysfunction has recently been shown to be predicted by nadir creatinine as well [59], emphasizing that a more severe manifestation affects both bladder and renal function. Urinary tract infections (UTI) can result from bladder dysfunction or vesicoureteral reflux, and can scar kidney tissue through inflammatory processes. Two studies were able to show that the number of urinary tract infections worsens renal prognoses [60,61], while another study was not able to prove any influence of UTI [62]. In a recent randomized controlled trial that compared antibiotic prophylaxis with and without circumcision in PUV patients, it was shown that the risk for febrile urinary tract infections could significantly be decreased by circumcision [63]. Rigid management of bladder dysfunction should also be performed in PUV patients with renal transplantation. Two recent studies showed that patients with Mitrofanoff procedures or clean intermittent catheterizations had significantly better graft survival rates, which was also comparable to graft survival rates for other indications [64,65]. Unsurprisingly, the sonomorphologic hallmarks of hypodysplastic kidneys such as hyperechogenicity, low kidney volume and pathologic corticomedullary differentiation also are prognostic factors for the progression towards ESKD [47]. A renal parenchymal volume above 88 mL/m^2^ of body surface excluded progression to ESKD, with a negative predictive value of 94.4% [47]. Hence, as a non-invasive, widely available tool, ultrasound should be used in addition to nadir creatinine in order to assess the ESKD risk in children with PUV.

Proteinuria is a common marker in nephrology that is used to assess kidney damage. Several studies have identified proteinuria as a negative prognostic marker for renal outcome in PUV patients [36,62,66,67]. Some studies have shown that urinary markers of inflammation and fibrotic remodelling can predict renal outcome. Transforming growth factor-β (TGF-β) is a marker of fibrosis, and is elevated in patients with an unfavourable renal outcome [67,68,69]. Monocyte chemotactic protein (MCP-1) recruits macrophages and is thus a marker of inflammation. Urinary MCP-1 was shown to predict ESKD in 20 patients with PUV [69]. Although molecular diagnostic tools such as proteomics have become more widely available and by far exceed the capabilities of single marker analyses, postnatal proteomics data in PUV patients are still missing. It can be expected that proteins originating from pathways of inflammation, cell death and fibrosis will be found in the urine of PUV patients. Further research on this topic can not only help to better predict clinical outcomes, but also identify new pathways that are involved in the progression of CKD; these are urgently needed to develop novel treatment strategies.

Recently, approaches that use artificial intelligence (AI) to predict clinical outcomes have received more attention and show promising results. Such tools rely on large data sets and multiple parameter entry using machine learning (ML), in order to predict the course of a disease. For example, in glomerular disease such as IgA-nephropathy, such a tool is very advanced, and is even recommended in the 2021 KDIGO guidelines for adults [70,71]. This tool has already been modified for children from a data set of 1060 children [72]. In CAKUT diseases, AI models are still rare [73,74,75]. Very recently, the first ML model to predict outcomes in PUV has been proposed and made available as an online tool [76]. The model was created on the basis of 103 patients in Toronto and validated in a different center with 22 patients. The entry parameters were baseline eGFR, nadir serum creatinine, grade of vesicoureteral reflux and renal hypodysplasia. Using those parameters, the risks of CKD, ESKD and clean intermittent catheterization (CIC) were predicted with c-indices of 0.78, 0.89 and 0.64, respectively. Interestingly, in this model renal dysplasia was the most relevant parameter for CKD prediction, while the well-known nadir creatinine was most relevant for ESKD prediction. Given the limitations of this model, with its relatively small training group and the subjective nature of some of its parameters and outcomes, it demonstrates the feasibility of AI to predict the course of PUV patients, and therefore supports the need for international registries with large data sets to further improve the predictive capacities of the models. 

## 5. Management of Chronic Kidney Disease in Posterior Urethral Valves

**Figure 3:**
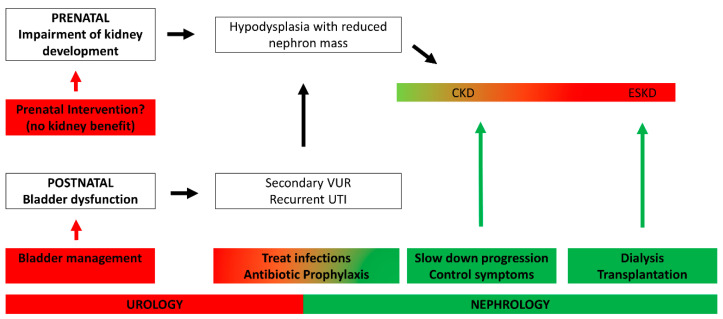
**Interventions to prevent and treat chronic kidney disease in posterior urethral valves.** Prenatal intervention with vesicoamniotic shunting or fetal cystoscopy yields no renal benefit. Bladder dysfunctions should be treated because they increase the risks of developing chronic kidney disease (CKD). Urinary tract infections (UTI) should be treated, and possibly a prophylaxis should be administered. All boys should be regularly seen by a pediatric nephrologist, in order to assess possible CKD. CKD progression should be slowed down, and CKD symptoms should be treated. In end-stage kidney disease (ESKD), dialysis or preferably transplantation is necessary.

Management of CKD in PUV patients is interdisciplinary, and requires both a pediatric urologist and a pediatric nephrologist. The possible interventions as well as their share among those disciplines are summarized in Figure 3. Bladder dysfunction should be treated by a specialized urologist and include pharmacotherapy (anticholinergic substances or ⍺-blockers), training in different voiding techniques (e.g., timed voiding or double voiding), CIC with or without nocturnal drainage and ultimately surgical procedures with correction of secondary vesicoureteral reflux, bladder augmentation or Mitrofanoff procedures. When kidney function is impaired, the general principles of CKD treatment should be applied in patients with PUV in addition to the urological management. The main goal is to slow down progression of CKD by controlling hypertension and proteinuria, the two major accelerators of loss of kidney function, and to avoid infections. If present, both hypertension and proteinuria can be addressed with the use of renoprotective ACE inhibitors, which decrease arterial blood pressure by RAAS inhibition and reduce proteinuria by a reduction in intraglomerular pressure. Furthermore, ACE inhibitors reduce renal remodelling. The second goal in CKD treatment is to control the symptoms, such as electrolyte and acid base disorders, anemia and metabolic bone disease, and to ensure adequate nutrition and growth.

### 5.1. Prenatal Intervention Does Not Prevent CKD

Since irreversible damage to both the kidneys and the bladder occurs in utero, there have been proposals to intervene as early as possible during pregnancy. The most common technique used is the placement of an intrauterine vesicoamniotic shunt (VAS); another option is fetal cystoscopy. Both approaches seem to be comparable in outcome [77]. In 2013, the PLUTO trial, the only prospective, randomized trial, analyzed the outcomes of VAS in 31 pregnancies [78]. With seven complications in six fetuses, the complication rates were high. There was a small benefit in the short-term survival of the 16 VAS patients, but no benefit in renal outcome was observed. A meta-analysis of nine studies showed that intrauterine intervention did not significantly improve survival at 6 to 12 months or at 2 years, and demonstrated no renal benefit in 112 fetuses treated with VAS versus 134 conservatively managed babies [79]. Kohl et al. recently showed that VAS before the 16th week of gestation resulted in a normal renal outcome in 15/19 survivors in the short-run, while survivors of VAS after completion of the 16th week of gestation showed normal renal function in only 9/28 [80]. Consequently, the authors argue that very early intervention might be the key to achieve better renal outcomes. In severe LUTO phenotypes, early intervention can be life saving for the fetus, as it attenuates pulmonary hypoplasia, which is the main cause of mortality in PUV fetuses and neonates. Therefore, fetuses with a severe phenotype and early anhydramnion might have an indication for VAS to ensure their survival. Overall, the data existing today indicate that developmental kidney impairment, beginning with PUV formation in the fifth week of gestation [10], cannot be reversed; furthermore, long-term survival cannot be improved by intrauterine shunting. It should therefore be conducted with caution. Further studies are needed to optimize prenatal care and selection of patients who might benefit from fetal intervention.

### 5.2. Optimal Bladder Control Leads to Improved Graft Survival in Kidney Transplantation

Patients with CAKUT etiology for renal transplantation have better graft survival as well as patient survival rates than patients with other acquired reasons for kidney transplantation [81]. This is possibly due to lower cardiovascular comorbidities as well as the absence of the risk of disease recurrence. However, among CAKUT patients, PUV patients were shown to perform worse [82,83,84]; this was possibly due to the bladder comorbidity, which might not ensure low-pressure storage and complete emptying. By contrast, in a newer cohort with lower urinary tract malformations, patients did not perform worse than patients with upper urinary tract malformations [85]. This might be due to the better care received for bladder dysfunction. Saad et al. found no difference in renal function after transplantation in LUTO patients and others, as long as intravesical pressure was adequately reduced. In a French multicenter study, Marchal et al. found that even though enterocystoplasties and continent urinary diversions exposed grafts to more frequent acute graft pyelonephritis, patient and graft survival rates in lower urinary tract malformations at 10 years were similar to the survival rates of other kidney transplantations on native bladders [65]. Amesty et al. recently showed that optimal bladder control using either clean intermittent catheterization or Mitrofanoff procedures resulted in more urinary tract infections, but prolonged graft survival [64]. It should therefore be mandatory to screen for and treat bladder dysfunction before renal transplantation, in order to prolong graft survival and ensure the optimal use of scarce transplant organs.

## 6. Conclusions

Chronic kidney disease in boys with PUV is caused by impaired intrauterine kidney development, which results in hypodysplastic kidneys with a lower nephron mass. Despite postnatal valve ablation, bladder dysfunction may persist and further decrease kidney function through urinary tract infections. Fetal urinary analysis as well as non-invasive fetal sonography can predict the risk of ESKD prenatally. The best postnatal predictor for progression to CKD is the nadir serum creatinine after valve ablation in infancy. Data on urinary markers beyond microalbuminuria are scarce, and novel postnatal biomarkers such as proteomic data are missing and should further be investigated.

Prenatal intervention yields no renal benefit. Therefore, the treatment options to mitigate CKD are optimal control of bladder dysfunction, slowing down the progression of CKD by avoiding infections, and treating hypertension and proteinuria. When transplantation is needed, graft survival depends on the degree of bladder dysfunction. Patients with optimal voiding strategies, e.g., with clean intermittent catheterization or Mitrofanoff procedures, show similar graft survival rates to patients with other indications for renal transplantation.

## Figures and Tables

**Table 1:** **Most relevant prenatal and postnatal risk factors for chronic kidney disease development in boys with posterior urethral valves.** ESKD (end-stage kidney disease), UTI (urinary tract infection).

Prenatal	Postnatal
**Ultrasound** Signs of hypodysplasia	**Ultrasound** Signs of hypodysplasia
**Fetal urine** β-2-microglobuline ↑, chloride ↑ 12PUV (peptide-panel)	**Nadir serum creatinine** <0.4 mg/dL: very low risk for ESRD >0.8–1.0 mg/dL high risk for ESRD
	**Presence of bladder dysfunction**
	**Recurrent febrile UTI**
	**Urinary markers** microalbuminuria TGF-β ↑, MCP-1 ↑

## Abbreviations

AI	artificial intelligence
CAKUT	congenital anomalies of the kidneys and urinary tract
CKD	chronic kidney disease
ESRD	end-stage renal disease
LUTO	lower urinary tract obstruction
ML	machine learning
PUV	posterior urethral valves
RRT	renal replacement therapy
TUI	transurethral incision
UTI	urinary tract infection
UUO	unilateral ureteral obstruction
VAS	vesicoamniotic shunt
